# Plant-Derived Compounds as a Tool for the Control of Gastrointestinal Nematodes: Modulation of Abamectin Pharmacological Action by Carvone

**DOI:** 10.3389/fvets.2020.601750

**Published:** 2020-12-16

**Authors:** M. V. Miró, S. Luque, P. Cardozo, M. Lloberas, D. M. Sousa, A. M. S. Soares, L. M. Costa-Junior, G. L. Virkel, Adrian L. Lifschitz

**Affiliations:** ^1^Centro de Investigación Veterinaria de Tandil (CIVETAN) (CONICET-CICPBA-UNCPBA), Facultad de Ciencias Veterinarias, Universidad Nacional del Centro, Tandil, Argentina; ^2^Laboratorio de Parasitología, Instituto Nacional de Tecnología Agropecuaria (INTA), Estación Experimental, Balcarce, Argentina; ^3^Laboratory of Parasite Control, Department of Pathology, Center for Biological and Health Sciences, Federal University of Maranhão, São Luis, Brazil; ^4^Laboratory of Plant Biochemistry, Department of Chemical Engineering, Center for Exact Sciences and Technology, Federal University of Maranhão, São Luis, Brazil

**Keywords:** carvone, abamectin, drug-interaction, resistant nematodes, P-glycoprotein

## Abstract

The combination of synthetic anthelmintics and bioactive phytochemicals may be a pharmacological tool for improving nematode control in livestock. Carvone (R-CNE) has shown *in vitro* activity against gastrointestinal nematodes; however, the anthelmintic effect of bioactive phytochemicals either alone or combined with synthetic drugs has been little explored *in vivo*. Here, the pharmacological interaction of abamectin (ABM) and R-CNE was assessed *in vitro* and *in vivo*. The efficacy of this combination was evaluated in lambs naturally infected with resistant gastrointestinal nematodes. Additionally, the ligand and molecular docking of both molecules to P-glycoprotein (P-gp) was studied *in silico*. The presence of R-CNE produced a significant (*p* < 0.05) increase of Rho123 and ABM accumulation in the intestinal explants. After 60 min of incubation, Rho123 incubated with R-CNE had a 67 ± 21% higher concentration (*p* < 0.01) than when it was incubated alone. In the case of ABM, a significant increase in the intestinal concentrations was observed at 15 and 30 min after incubation with R-CNE. In the *in vivo* assay, no undesirable effects were observed after the oral administration of R-CNE. The coadministration of the natural compound prolonged ABM absorption in lambs. ABM *T*_½_ absorption was 1.57-fold longer (*p* < 0.05) in the coadministered group. Concentrations of R-CNE between 420 and 2,593 ng/mL were detected in the bloodstream between 1 and 48 h posttreatment. The *in vivo* efficacy of ABM against gastrointestinal nematodes increased from 94.9 to 99.8% in the presence of R-CNE, with the lower confidence interval limit being >90%. *In vitro*/*in vivo* pharmacoparasitological studies are relevant for the knowledge of the interactions and the efficacy of bioactive natural products combined with synthetic anthelmintics. While ADMET (absorption, distribution, metabolism, excretion, and toxicity) predictions and the molecular docking study showed a good interaction between ABM and P-gp, R-CNE does not appear to modulate this efflux protein. Therefore, the pharmacokinetic–pharmacodynamic effect of R-CNE on ABM should be attributed to its effect on membrane permeability. The development of pharmacology-based information is critical for the design of successful strategies for the parasite control.

## Introduction

Gastrointestinal nematodes cause one of the main diseases affecting livestock in grazing systems worldwide (1, 2). Despite the diverse chemical groups available to control parasitic diseases in ruminants, macrocyclic lactones (MLs) have been the most widely used drugs during the last 30 years (3). The intensive and injudicious use of these compounds has led to therapeutic failures and the expansion of anthelmintic resistance (4). Considering the high level of resistance to MLs, there is an urgent need to search for novel strategies to extend the life span of antiparasitic agents. One possibility of maintaining the use of already existing drugs is by combining them with unusual compounds with the aim to reduce the biochemical mechanisms of resistance (5). Different plant-derived products were tested as potential tools for the treatment of helminth infestations in ruminants (6, 7), which can contribute with additive or synergistic effects when interacting with antiparasitic drugs. R(-) carvone (R-CNE) (5-isopropenyl-2-methyl-2-cyclohexenone) occurs naturally as dextrorotatory (d-) and levorotatory (l-) enantiomers in several food items such as mint and caraway. R-CNE is also used as pesticide and, together with other active substances, as a zootechnical feed additive (8). There is scarce information about the anthelmintic use of this compound. The *in vitro* activity of R-CNE has been shown, particularly against *Haemonchus contortus* egg hatch. The 50% lethal concentration of CNE was 85 μg/mL, and this effect was increased after the combination with different phytochemicals (7). Moreover, the effect of the long-term administration of encapsulated R-CNE was recently evaluated in sheep, and a significant reduction in the fecal egg count was obtained (9). These promissory observations show the need for additional research on the potential of this phytochemical compound as a pharmacological alternative to treat gastrointestinal parasitic diseases of ruminants. Overall, there is a need to gain further knowledge on the potential of this phytochemical compound to increase the antiparasitic action of synthetic anthelmintics upon their combined administration. Thus, bioactive phytochemicals may contribute to an increase in parasite control by pharmacokinetic and/or pharmacodynamic interactions, enhancing the effect of existing anthelmintic drugs.

MLs such as abamectin (ABM) are well-known substrates and/or inhibitors of the ATP- binding cassette (ABC) transporters, such as P-glycoprotein (P-gp) (10, 11). This transmembrane protein is able to pump out several unrelated endobiotics and xenobiotics from mammal and parasite cells through an ATP-dependent process (12). Many natural compounds from plant sources inhibit P-gp and increase the effectiveness of conventional chemotherapy without undesirable toxicological effects (13). It has been also well documented that phytochemicals may affect intestinal absorption, with further effects on the pharmacokinetics of different compounds through several mechanisms (14). In this context, it is necessary to assess whether the combined administration of R-CNE and ABM increases the anthelmintic effect and if a beneficial drug–drug interaction occurs. Thus, this work assessed *in vitro* and *in vivo* the pharmacological interaction of ABM and R-CNE. The efficacy of this combination was evaluated in lambs naturally infected with resistant gastrointestinal nematodes. Additionally, the ligand and molecular docking of both molecules to P-gp was studied *in silico*.

## Materials and Methods

Animal procedures and management protocols were carried out according to internationally accepted animal welfare guidelines (15) and approved by the Animal Welfare Committee of the Faculty of Veterinary Medicine, Universidad Nacional del Centro de la Provincia de Buenos Aires, Tandil, Argentina (Internal Protocol: FCV-UNCPBA 11/2018; approval date: August 27, 2018).

### *In vitro* Evaluation of Intestinal Accumulation

The modulation of intestinal accumulation by R-CNE was assessed using the intestinal explant model. Cattle ileum samples were obtained from a local slaughterhouse (Mirasur SA, Tandil, Argentina) located 16 km away from the laboratory facilities. Ileum samples were obtained from Aberdeen Angus/Hereford crossbreed steers of approximately 350 kg in weight; immediately after extraction, samples were rinsed gently with ice-cold KCl 1.15% and Euro-Collins solution (0.19 M glucose; 15.43 mM KH_2_PO_4_; 42.48 mM K_2_HPO_4_; 15.02 mM KCl; 10 mM CO_3_HNa), and conserved in Euro-Collins solution at 4°C during transport to laboratory facilities. The incubation process was started immediately after obtaining the tissue. Intestinal explants were prepared as described below, at 0–4°C. Cylindrical explants (average weight 211.2 ± 56.1 mg, 1-cm diameter) were obtained from the ileum tissue and cultured in well plates containing 6 mL of Williams' medium E (WME). The plates were incubated in an orbital shaker (Ferca, Buenos Aires, Argentina) set at 60 rpm and maintained at 37°C under a humidified atmosphere of 95% O_2_:5% CO_2_. During preparation, explants were preincubated for 20 min to slough off any dead cells. During this period, ileum explants were preincubated in the presence or absence of 3.33 mM of R-CNE or 0.5 μM of ivermectin (IVM), which was used as positive control because IVM is a potent P-gp inhibitor (16, 17). After the preincubation period (*t* = 0), WME was completely replaced with fresh medium fortified with 0.5 μM of Rhodamine 123 (Rho123) or 0.05 μM of ABM as substrates. Thus, both substrates were incubated alone (control assays) or in the presence of R-CNE or IVM. The plates were incubated for 15, 30, 45, or 60 min. After the incubation period, the explants were removed, carefully washed with NaCl 0.9%, dried, weighed, and immediately stored at −20°C until analysis.

To determine Rho123 and ABM concentrations within intestinal explants, samples were homogenized with 0.5 mL of methanol (Rho123) or 0.5 mL of acetonitrile (ABM). Homogenates were mixed with a high-speed shaker (Multi Tube Vortex; VWR Scientific Products, West Chester, PA) at room temperature for 15 min. Then, the mixtures were centrifuged at 2,000 g at 4°C for 10 min, and the supernatant was manually transferred to a clean tube. Samples of Rho123 were mixed with 2.5 mL of NaCl 0.9% to reach a final volume of 3 mL. Rho123 concentrations in intestinal explants (16 replicates per sampling time) were determined in a fluorescent spectrophotometer RF-5301PC (Shimadzu Corporation, Kyoto, Japan) set at an excitation wavelength of 485 nm and an emission wavelength of 520 nm (18). The ABM concentrations in explants (8–14 replicates per sampling time) were determined by high-performance liquid chromatography (HPLC), following the technique described in section *in vivo* Pharmacokinetic and Efficacy Study.

Tissue viability was assessed by estimating lactate dehydrogenase (LDH) leakage. This enzyme activity was measured both in the culture medium and in explants at each incubation time in a spectrophotometer (T80+UV/Visible Spectrometer; PG Instruments Limited, Lutterworth, England), following the method described by Moldeus et al. (19). Percentages of LDH leakage were calculated as follows:

LDH leakage (%):

$$\begin{array}{l} =\frac{Activity\;in\;culture\;medium}{\left(Activity\;in\;culture\;medium+activity\;in\;explant\right)}\;\times \;100 \end{array}$$

where enzyme activities in culture medium or in explants are expressed as mmol of NADH oxidized per minute in the presence of the sodium pyruvate as substrate.

### *In vivo* Pharmacokinetic and Efficacy Study

The trial involved 28 Corriedale and Texel crossbreed lambs (22–42 kg) naturally infected with resistant gastrointestinal nematodes. The trial was conducted in a sheep experimental unit (Estación Experimental INTA, Balcarce, Argentina) where a parasite control program based on the intensive use of antiparasitic drugs has been implemented during many years, leading to anthelmintic resistance to MLs and benzimidazoles. Animals were selected based on worm egg per gram counts (epg), using the modified McMaster technique with a sensitivity of 10 epg (20). Experimental animals had an average of 2,172 ± 1,002 epg counts, ranging from 580 to 3,885. Animals were placed in a paddock and fed hay *ad libitum* together with commercial concentrate feed. All the animals had free access to water. Lambs were assigned to three experimental groups. Group A received ABM (Necaverm® Rosenbusch, Argentina) (single dose of 0.2 mg/kg, orally) (*n* = 10). Group B received ABM (single dose of 0.2 mg/kg, orally) coadministered with R-CNE (Euma, Argentina) (100 mg/kg, four oral doses administered every 24 h) (*n* = 10). The first dose of R-CNE was administered 20 min before the single ABM administration, and then it was repeated every 24 h until the dosing schedule was completed. The lambs of group C included untreated controls (*n* = 8). The efficacy of each treatment was characterized by collecting fecal samples from all the lambs in each experimental group on days −1 and 14 posttreatment for epg count estimation. For the plasma disposition study (*n* = 8 in groups A and B), jugular blood samples (2 mL) were collected into heparinized Vacutainer tubes before treatment and at 2, 4, 6, 8, 24, 28, 48, 52, 72, 76, 96, and 168 h posttreatment. Blood samples were centrifuged at 2,000 *g* for 15 min; the recovered plasma was kept in labeled vials and stored at −20°C until the analysis of ABM and R-CNE plasma concentrations by HPLC.

ABM was extracted from plasma following the technique described by Lifschitz et al. (21). Briefly, 0.5 mL of plasma was fortified with 20 ng/mL of the internal standard moxidectin. ABM was extracted by adding 1 mL of acetonitrile and then mixed with a high-speed shaker (Multi Tube Vortex; VWR Scientific Products, West Chester, PA) for 15 min. The mixture was centrifuged at 2,000 *g* at 4°C for 10 min, and the supernatant was manually transferred to a clean tube, evaporated to dryness, derivatized (22), and analyzed by HPLC. Fluorescent detection was performed using a spectrofluorometric detector (RF-10; Shimadzu) set at an excitation wavelength of 365 nm and an emission wavelength of 475 nm. The mobile phase was composed of 0.2% acetic acid (in water)–methanol–acetonitrile (1.6:60:38.4; vol/vol/vol). The flow rate was set at 1.5 mL/min through a reverse-phase C18 column (Kromasil; Eka Chemicals, Bohus, Sweden).

R-CNE analysis was performed according the technique described by Tao and Pereira (23), with modifications. Aliquots of plasma (0.25 mL) were mixed with acetonitrile, agitated and centrifuged. Supernatants were injected into the HPLC system fitted with a Kromasil C18 column. The mobile phase was water/acetonitrile (40/60) at an isocratic flow of 1.3 mL/min. R-CNE was analyzed using a UV detector (Shimadzu, SPD-10A).

The fecal samples were analyzed to obtain the epg counts. The coprocultures were prepared with 10 g of feces from a pool of each experimental group obtained on days−1 and 14. The nematode genera and species were identified through the third-stage larvae recovered from the coprocultures (24). The fecal egg count reduction test (FECRT) was calculated using the Abbott's formula (25), with modifications:

$$\begin{array}{l} FECRT\left(\%\right)=100\times \left(1-\frac{T2}{T1}\;\times \;\frac{C1}{C2}\right) \end{array}$$

where *T*1 and *T*2 are the arithmetic mean epg counts in the treated group on days 0 and 14, respectively, and *C*1 and *C*2 are the arithmetic mean epg counts in the control group on days 0 and 14, respectively. The 95% confidence intervals were calculated following Coles et al. (26).

### *In silico* Evaluation of ABM and R-CNE Interaction

The characteristics of the absorption, distribution, metabolism, excretion, and toxicity (ADMET) prediction and drug-like properties of the ligands ABM and R-CNE were determined using the preADMET online software tool [https://preadmet.bmdrc.kr/adme/ (27)].

The binding of ABM and R-CNE to P-gp from *Caenorhabditis elegans* (Cel-Pgp-1) (28) was assessed *in silico* by molecular docking. The three-dimensional structures of R-CNE (CID 439570) and ABM (CID 6434889) were obtained from PubChem (https://pubchem.ncbi.nlm.nih.gov/), in .sdf format. The structures were optimized using force field MMFF94, incorporated into a built-in geometry optimization algorithm of MarvinSketch, ChemAxon (https://chemaxon.com/products/marvin). The cavity of Cel-Pgp-1 where ABM was predicted to bind (29) was chosen for molecular docking of ABM and R-CNE.

The Cel-Pgp-1 structure, at a resolution of 3.40 Å, was obtained from PDB (https://www.rcsb.org/structure/4F4C), in .pdb format. As part of the preparation of the Cel-Pgp-1 file for docking, the associated ligands were removed using Chimera, version 1.13.1 (https://www.cgl.ucsf.edu/chimera/). The software Autodock 4 (release 4.2.6) was used for molecular docking experiments. The grid was built following David et al. (29), with modifications. The size of the binding area was centered in the inner cavity of Cel-Pgp-1 at the point *x* = −20.8, *y* = 6.9, *z* = −9.5 Å. The Lamarckian genetic algorithm was used for docking simulation, and all the other parameters were set at the default value (29). The 10 generated poses were assigned a score calculated by AutoDock, which can be considered as the estimated free energy of ligand binding. Results are expressed in kcal/mol. The lowest-energy conformations of the ligands are presented.

## Data Analysis

Data are expressed as mean ± standard deviation (SD). Rho123 accumulation in ileum explants is presented as pg/mg of tissue; *in vitro* accumulation rates of this P-gp substrate in gut wall are shown as pg/min per mg of tissue. The plasma concentration-vs.-time curves obtained after treatment of each animal were fitted with the PK Solutions 2.0 (Ashland, OH, USA) software. Pharmacokinetic parameters were determined using a non-compartmental model method (30). The statistical analysis was performed using the Instat 3.0 software (GraphPad Software, CA, US). Depending on the experiment, Rho123, ABM, and R-CNE concentrations; pharmacokinetic parameters; and epg counts were statistically compared using Student *t*-test, Mann–Whitney *U*-test or Kruskal–Wallis test. Differences were considered statistically significant at *p* < 0.05.

## Results

### *In vitro* Intestinal Accumulation

Tissue viability was assessed through LDH leakage. LDH activity was monitored in the culture medium and in explants from bovine ileum during 90 min of culture. Enzyme activities were used to estimate mean percentages of LDH released by the explant to the medium relative to the total enzyme activity. The percentages of LDH released from the explants to the culture medium were low, indicating a good viability of the explants during the incubation period. Mean (±SD) percentages of LDH leakage to the incubation medium were 2.13 ± 1.16% (15 min), 6.16 ± 1.76% (45 min), and 7.11 ± 2.37% (90 min).

**Figure 1:**
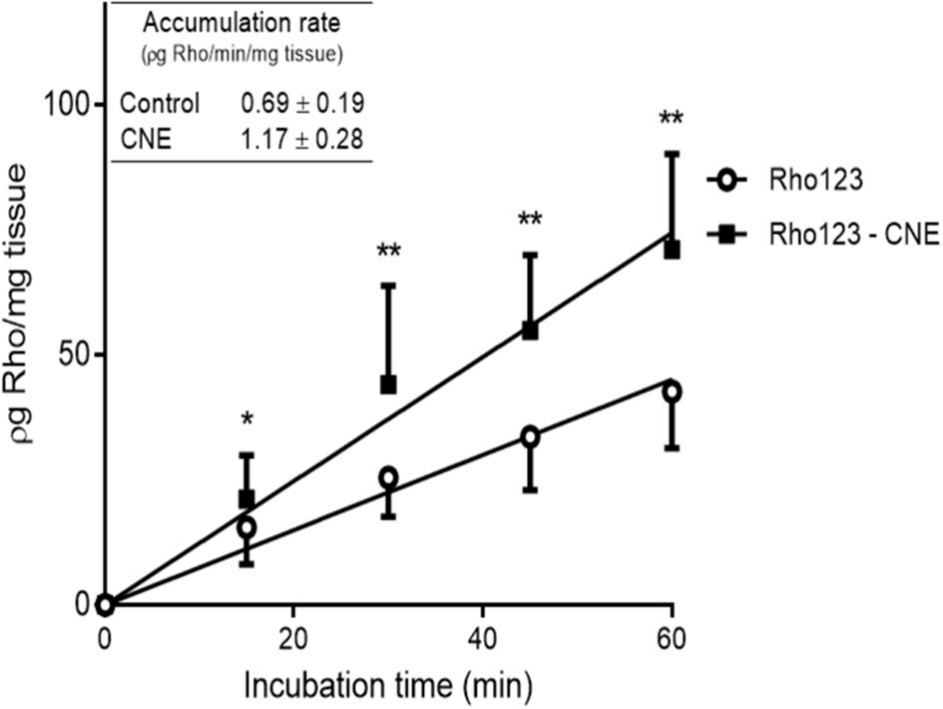
Accumulation of Rhodamine 123 (Rho123) in bovine ileal explants after incubation in absence or presence of R-carvone (R-CNE). The inserted table shows the rates of accumulation of Rho123 (ρg Rho/min/mg tissue). Data are the mean (±SD) of 16 determinations. Significantly different from control incubations (Rho123 alone) at *p < 0.05 and **p < 0.01.

**Figure 2:**
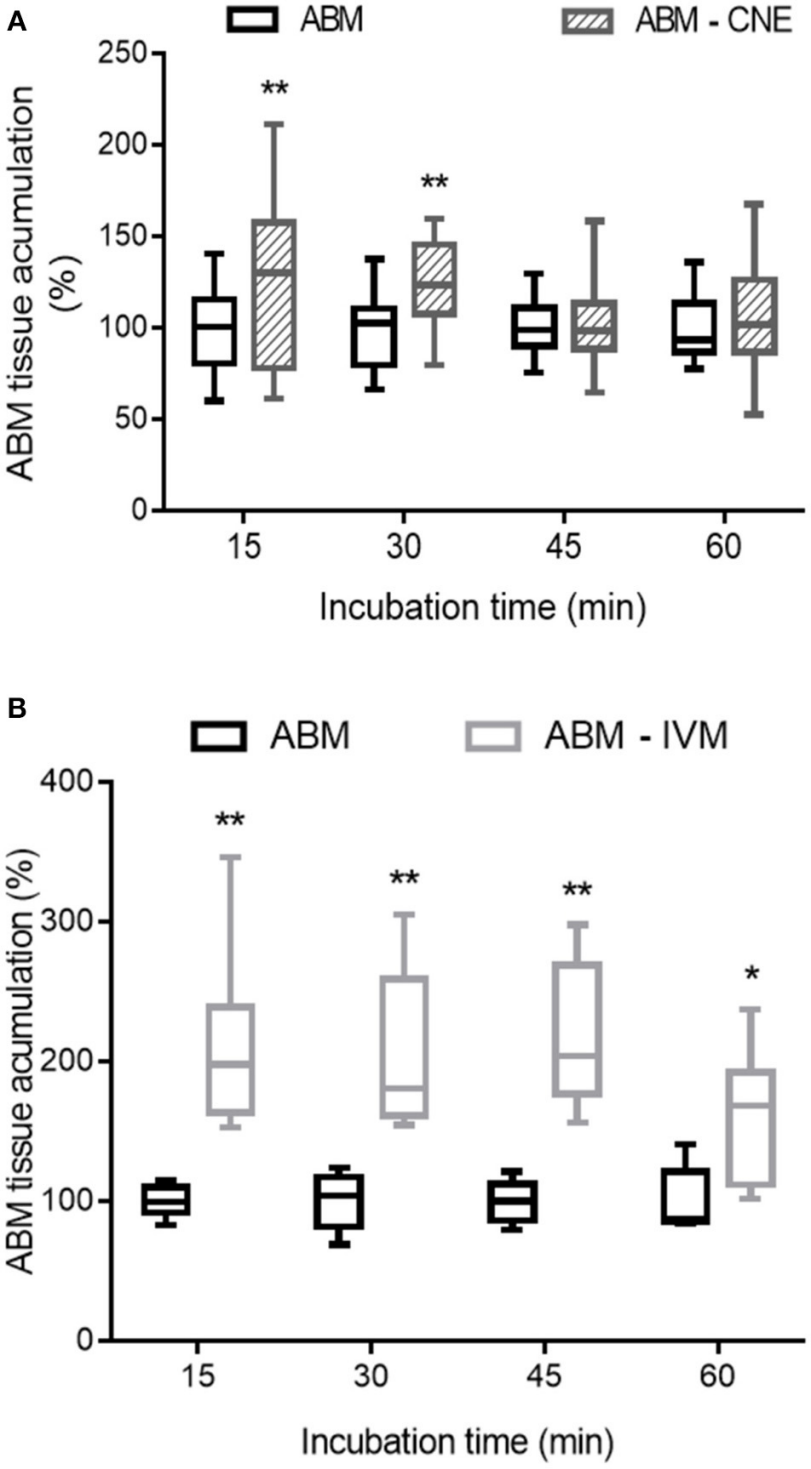
Effect of R-carvone (**A**, R-CNE) and ivermectin (**B**, IVM) on abamectin (ABM) accumulation in bovine ileal explants. Data are the mean (±SD) of 8–14 determinations and are expressed as the percentage of control group (ABM alone). Significantly different from control incubations at *p < 0.05 and **p < 0.01.

The presence of R-CNE significantly increased Rho123 concentrations in the intestinal explants at all incubation times (Figure 1). After 60 min of incubation, Rho123 concentration was 67 ± 21% higher (*p* < 0.01) in the explants incubated with R-CNE. Thus, Rho123 accumulation rate was significantly higher (*p* < 0.001) in the intestinal explants coincubated with R-CNE. The presence of R-CNE increased the intestinal concentration of the synthetic anthelmintic ABM at 15 and 30 min of incubation (Figure 2A). At 30 min of incubation, the intestinal concentration of ABM was 28.1 ± 35.7% higher in the presence of R-CNE than in the control. The modulation of the ABM intestinal accumulation was also evaluated using a well-known P-gp inhibitor, IVM (Figure 2B). The presence of IVM significantly increased the accumulation of ABM in the intestinal explants at all incubation times assessed (15, 30, 45, and 60 min).

### *In vivo* Pharmacokinetic and Efficacy Study

**Figure 3:**
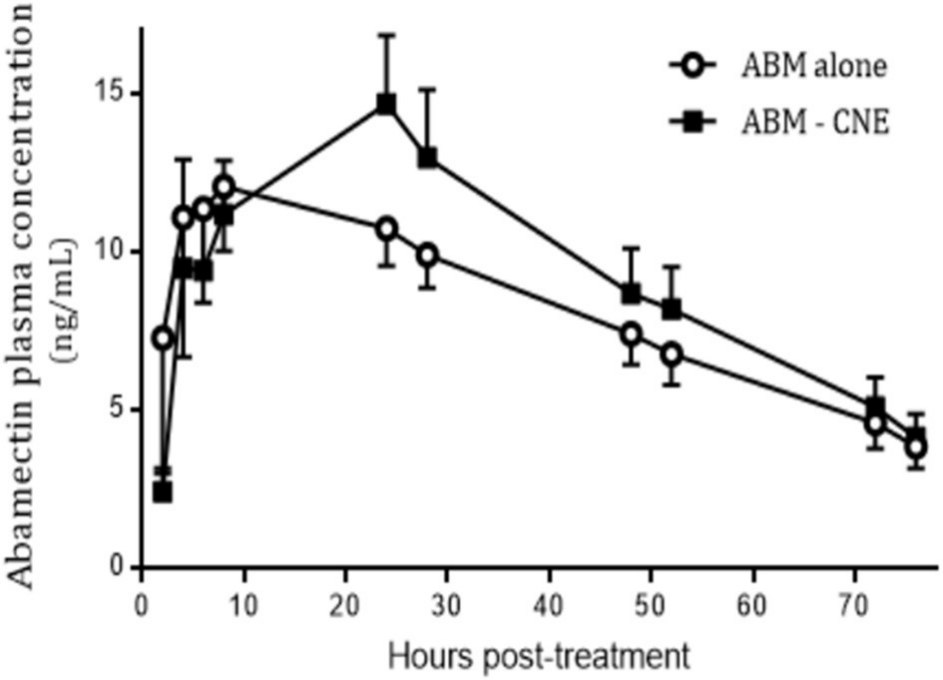
Plasma concentration profiles of abamectin (ABM) after its oral administration at 0.2 mg/kg (recommended dose) either alone or coadministered with R-carvone (R-CNE) (four doses of 100 mg/kg each) to lambs (*n* = 8) infected with resistant nematodes. Profile is shown up to 70 h posttreatment to focus on the effect of R-CNE on ABM absorption phase.

In the *in vivo* assay, no undesirable effects were observed after the oral administration of R-CNE. ABM was recovered from plasma up to 7 days posttreatment in both treated groups. The comparison of ABM plasma concentration profiles obtained after ABM administration either alone or coadministered with R-CNE in infected lambs is illustrated in Figure 3. The comparative pharmacokinetic parameters obtained after the administration of ABM or ABM+R-CNE are shown in Table 1. Concomitant administration with R-CNE prolonged ABM absorption in lambs. ABM *T*_½_ ab (absorption half-life) was 1.57-fold longer after the coadministration with R-CNE. A fast absorption and elimination of R-CNE were observed after its oral administration to lambs. The mean *C*_max_ was 1792 ± 598 ng/mL and was achieved at 2.75 ± 1 h after the administration of each dose.

**Table 1:** Comparative plasma pharmacokinetic parameters for abamectin (ABM), obtained after its oral administration (0.2 mg/kg) either alone or coadministered with R-carvone (R-CNE, four doses of 100 mg/kg every 24 h) to lambs.

**Kinetic parameters**	**ABM**	**ABM + R-CNE**
*C*_max_ (μg/mL)	16.20 ± 10.2^a^	17.15 ± 8.43^a^
*T*_max_ (h)	10.75 ± 9.6^a^	20.00 ± 8.82^a^
*T*_½_ ab (h)	4.12 ± 2.2^a^	6.48 ± 1.74^b(p = 0.031)^
*T*_½_ el (h)	28.04 ± 8.1^a^	24.53 ± 3.19^a^
AUC_0−168h_ (μg h/mL)	815.67 ± 303.1^a^	909.37 ± 405.86^a^

Data are expressed as mean ± SD (n = 8 animals).

C_max_, peak plasma concentration; T_max_, time to peak plasma concentration; *T*_½_ ab, absorption half-life; *T*_½_ el, elimination half-life; AUC_0−168h_, area under concentration vs. time curve from time 0 to the last concentration detected.

Different lowercase letters between ABM and ABM+R-CNE treatments mean statistical differences at p < 0.05.

*The values without significant differences have the similar letter (a). In the case that exist statistical differences the letters are different (a and b)*.

The fecal egg counts obtained for all experimental groups, including the results of the FECRT and upper and lower confidence limits (95%), are shown in Table 2. The efficacy after the ABM alone treatment was 94.9%, with the lower limit below 90%, and increased to 99.8% in the presence of R-CNE, with the lower confidence interval limit >90%. The main genera involved before the treatments were *Haemonchus* spp. (1%), *Teladorsagia* spp. (40%), *Trichostrongylus* spp. (32%), *Cooperia* spp. (12%), and *Chabertia* spp. (15%). After ABM treatment, the proportion of the genera was 88% for *Haemonchus* spp. and 12% for *Teladorsagia* spp. After the ABM+R-CNE administration, the distribution was 52% for *Haemonchus* spp., 32% for *Teladorsagia* spp., and 16% for *Trichostrongylus* spp.

**Table 2:** Mean (±SD) egg per gram (epg) counts and reduction of fecal egg counts (FECR) obtained 14 days after the oral administration of abamectin (ABM) given alone or combined with carvone (R-CNE) to naturally infected lambs.

**Experimental group**	**Mean epg counts**		**FECR (LCL-UCL)**
	**Day 0**	**Day 14**	**(%)**
Control	1,858 ± 1,123	750 ± 607	—
ABM	2,193 ± 858	45 ± 121	94.9 (65.0-99.0)
ABM + R-CNE	2,401 ± 1,072	2 ± 6.32	99.8 (98.2-99.9)

*LCL, lower confidence limit; UCL, upper confidence limit*.

### *In silico* Evaluation of ABM and R-CNE Interaction

The quantitative and qualitative results of the ADMET predictions for ABM and R-CNE are presented in Table 3. The binding energy/Gibbs energy (lowest-energy position identified) for ABM and R-CNE was −9.33 and −5.95 kcal/mol, respectively. The main residues responsible for the binding and stability of ABM were Tyr914, Arg916, Gly1032, Phe1033, Thr1035, and Pro1039. The key residues predicted for Cel-Pgp-1 interaction with R-CNE were Leu25, Phe323, Gln327, Thr760, Ala761, Gly764, Gly765, Ile767, Tyr768, Gln807, Cys810, Ser811, Met814, Thr876, and Thr879 (Supplementary Figure S1).

**Table 3:** Predicted ADMET properties of compounds abamectin (ABM) and R-carvone (R-CNE).

**ID**	**ABM**	**R-CNE**
**Absorption**		
Caco-2	50.900	477.420
MDCK	0.043	975.458
HIA	95.35	100
Pgp inhibition	Inhibitor	No
PWS	0.1100	636.470
PPB	88.90	58.040
Skin permeability	−2.630	−140.340
SKlog*P* value	5.106	2.599
**Distribution**		
BBB	0.221	106.060
**Metabolism**		
CYP 2C19 inhibition	Inhibitor	Inhibitor
CYP 2C9 inhibition	Inhibitor	Inhibitor
CYP 2D6 inhibition	No	No
CYP 2D6 substrate	No	No
CYP 3A4 inhibition	Inhibitor	No
CYP 3A4 substrate	Substrate	Substrate
**Toxicity**		
Algae at	0.000	0.056
Ames test	Non-mutagen	Mutagen
Carcino mouse	Positive	Negative
Carcino rat	Positive	Positive
Daphnia at	0.003	0.222
hERG inhibition	Ambiguous	Low risk

*Algae At, algae test; Ames test, Ames Salmonella; BBB, blood–brain barrier (C.brain/C.blood); Caco-2, Caco2-cell model; CYP, cytochrome P450; Carcino M, carcinogenesis test in the mouse; Carcino R, carcinogenesis test in rats; Daphnia At, test on crustacean daphnia; hERG Inhib., hERG-controlled potassium channel inhibition; HIA, human intestinal absorption model (HIA, %); MDCK, Madin–Darby canine kidney (nm/s); PGP, P-glycoprotein; PPB, plasma protein binding (%); PWS, pure water solubility (mg/L); Skin permeability, skin permeability (cm/h); SKlogP, calculated logP by SK atomic types*.

## Discussion

After more than 30 years of intensive use of MLs, the development of resistance is a seriously increasing problem in ruminants (31, 32). Plant-derived products may contribute to the improvement of parasite control by enhancing the effect of existing anthelmintic drugs. However, after a combined treatment, it is necessary to evaluate the potential drug–drug interactions between the administered compounds. As many bioactive phytochemicals may interact with P-gp (13), the R-CNE modulation of the intestinal accumulation of a known P-gp substrate was studied in the current work.

Ileum explants are an integrated system that offers diverse applications (33), such as evaluation of intestinal drug accumulation and *in vitro* modulation of efflux proteins. The methodology used here may be useful to establish an *in vitro/in vivo* correlation. LDH leakage is one of the parameters used to measure gut viability in *in vitro* experimental models of xenobiotic metabolism, transport, and/or toxicity. Increased extracellular LDH activity is expected after enterocyte plasma membrane damage, mainly when the intestinal tissue is exposed to potentially toxic drugs (34). Thus, LDH is a stable intracellular enzyme that can be readily detected when cell membranes are no longer intact (35, 36). Both extracellular and intracellular LDH activities were measured in the current work. The extracellular LDH activity accounted for <10% of the total (intracellular plus extracellular) enzyme activity, suggesting that the intestinal explants remain viable during the whole incubation period (90 min). Similarly, LDH leakage values <10% were recorded after 120 min of incubation of ileum explants from chicken (37).

Different pharmacological strategies have been evaluated to modify the pharmacokinetic behavior of the MLs and to improve their clinical efficacy. The involvement of P-gp in the excretion of ABM has been thoroughly investigated (10, 38, 39). *In vitro* methodologies using intestinal tissue or enterocytes demonstrated the influence of ABC transporters on the absorption and excretion of MLs (18, 40). In the current trial, the influence of R-CNE on the intestinal accumulation of Rho123, as specific P-gp substrate, was evaluated in ileum explants. The presence of R-CNE significantly increased Rho123 intestinal accumulation at all incubation times (Figure 1). After 60 min of coincubation, Rho123 concentration was 67% higher than with its incubation alone. Similarly, the absorption of Rho123 in sheep intestine fixed in Ussing chambers was significantly increased (69%) in the presence of IVM, a well-known P-gp inhibitor (18).

As a forward step, R-CNE modulation of ABM intestinal accumulation was studied. R-CNE significantly increased ABM accumulation in ileum only at 15 and 30 min of incubation (Figure 2A). However, when the modulation was assessed with the known P-gp inhibitor, IVM, intestinal concentrations of ABM increased at all the analyzed times (Figure 2B). IVM has shown high potency as P-gp inhibitor, equivalent to most potent inhibitors such as valspodar (10, 17); therefore, it was used as positive control in the intestinal explants assay. Although the interaction of MLs with other ABC transporters, such as MRP (16, 41) and BCRP (42), may confuse the interpretation of these results, it is clear that MLs are potent P-gp and rather weak MRP and BCRP inhibitors. The IVM concentrations that inhibited the activated ATPase activity by 50% were 2.5 μM for P-gp and between 9 and 40 μM for MRPs (16). In a different *in vitro* model, the concentrations of ABM necessary to inhibit P-gp transport were 3-fold lower than the necessary one to inhibit MRPs-mediated transport (41). In fact, using transfected canine kidney cells, no significant MRP1 and MRP2 avermectin transport was found in the presence of functional P-gp (43).

Although the P-gp–mediated transport of MLs is well-known, the potential interaction of R-CNE and ABM with P-gp should be thoroughly evaluated. Determining the crystal structure of Cel-Pgp-1 (28) is relevant to evaluate drug binding using molecular docking. Molecular docking studies have played a crucial role in computer-aided drug design (44). The interaction of both molecules with P-gp was evaluated through *in silico* studies in the current work. Although the use of *C. elegans* P-gp can be considered an experimental limitation if we intend to compare it with the transport in the host, it allows us to study drug–drug interactions at the parasite level. Additionally, as P-gp is a very conserved and ubiquitous protein and the conformation described for Cel-Pgp-1 is similar to that found for mammalian P-gp (29), the results of substrate recognition could be applied to the different animal models used in the current trial.

ADMET predictions showed that ABM would be a P-gp inhibitor, whereas R-CNE would not. The ADMET prediction also implies that ABM will have difficulty to cross the blood–brain barrier, whereas R-CNE may easily pass through it. The molecular docking results of this work indicated different interactions of ABM and R-CNE with Cel-Pgp-1. The binding affinity of drugs and drug candidates varies; however, in general, a design goal is engineering a compound with a binding affinity on the order of 0.1 nM, which is equivalent to a Gibbs energy of −14 kcal/mol (45). ABM showed a lower energy-binding value than R-CNE; this result is consistent with the specific binding of ABM to P-gp, as previously demonstrated (29). The binding site and binding energy of R-CNE in the current work were similar to those of the anthelmintic drug thiabendazole (29). These authors suggested that the transmembrane translocation of thiabendazole is unlikely. Accordingly, the R-CNE translocation would be also unlikely, and R-CNE would not be a P-gp modulator at the parasite level. Thus, the influence of R-CNE on drug absorption and accumulation may be explained with other mechanisms. Different bioactive phytochemicals may increase intestinal absorption by enhancing enterocyte membrane permeability or by opening paracellular tight junctions (14). In fact, R-CNE was used as penetration enhancer that allows drug permeation through the skin and enhanced the effect of a vasodilator drug after topical administration (46). Thus, the increased intestinal accumulation of Rho123 and ABM observed in the current trial may be supported by the effect of R-CNE on the membrane of intestinal cells.

Currently, numerous marketed pharmaceutical formulations contain the combination of two or three synthetic anthelmintics. However, there is scarce information of the combination of bioactive phytochemicals and antiparasitic drugs. The concomitant administration of sainfoin and IVM to sheep by the oral route decreased the absorption of the synthetic anthelmintic (47). In a recent study, the *in vivo* coadministration of the natural terpene thymol with albendazole also led to a negative pharmacokinetic interaction by significantly reducing the ruminal sulforeduction of the metabolite albendazole sulfoxide to the parent drug albendazole (48). The information obtained with the *in vitro* and *in silico* studies was complemented with the *in vivo* trial. The *in vivo* trial evaluated whether or not the BM+R-CNE combination would be a useful pharmacological tool, including the assessment of a potential drug–drug interaction between both compounds in naturally infected sheep. The *in vivo* coadministration of R-CNE and ABM prolonged the absorption half-life by 57% (Table 1). The ABM *T*_max_ tended to be delayed in the presence of R-CNE. Although P-gp modulation may enhance ML absorption, for example, after IVM oral coadministration with itraconazole in sheep (49), the *in silico* simulations demonstrated that a drug–drug interaction between R-CNE and ABM at the P-gp level is unlikely. Therefore, the beneficial effect of R-CNE on the pharmacokinetics of ABM may be based on the effect of the natural products on the enterocyte membrane permeability (14).

The plasma concentration profiles of R-CNE ranged between 420 and 2,593 ng/mL, and the phytochemical was eliminated rapidly after the administration of each dose. R-CNE is metabolized by phase I and phase II metabolism, which favors its rapid excretion (50). *In vitro* efficacy tests on *Haemonchus* spp. eggs and larvae have corroborated that the R-CNE concentrations necessary to obtain an efficacy >90% were 370 μg/mL (7), which is 142-fold higher than the *C*_max_ observed in the current trial. Given the low *in vivo* exposure of parasites to R-CNE, the compound would not be able to achieve a high anthelmintic efficacy if it were administered alone. The coadministration of R-CNE and ABM increased the anthelmintic efficacy from 94.9 to 99.8%; according to Coles et al. (26), the criteria to classify drug efficacy should change from suspected of resistance (ABM alone) to sensitive (ABM+R-CNE) (Table 2). This enhanced efficacy may be based on the pharmacokinetic interaction between R-CNE and ABM within the host and on a pharmacodynamic interaction related to the *in vivo* effect of R-CNE on the nematodes. The low level of resistance observed for the ABM alone treatment may be a limitation and may underestimate the impact of the treatment combined with R-CNE. The current trial was carried out on an experimental farm with a history of resistance to MLs and benzimidazoles. However, at the time of the *in vivo* trial, the low proportion of *Haemonchus* spp. within the initial parasite population could explain the increase in the FECRT. Further studies are needed to test this pharmacological tool under different resistance scenarios.

Another important issue is to establish the additive or synergistic feature of this type of combination. The *in vitro* evaluation of the binary combination of R-CNE with other bioactive phytochemicals, such as anethole, showed synergistic activity in the egg hatch assay with *H. contortus* eggs (7). However, the *in vivo* administration of R-CNE+anethole combination to infected lambs for 45 days resulted in a reduction in fecal egg counts of *H. contortus*, but there was no effect on total worm count (9). Proper dosing regimen and formulation are essential to ensure the best performance after the administration of a bioactive phytochemical to ruminants. From a practical point of view, the dosing schedule used for R-CNE in the current trial is a limitation and needs to be improved by the pharmaceutical technology. The challenge for the future use of monoterpenes as antiparasitic compounds is conditioned to suitable pharmaceutical formulations that provide sustained concentrations in the tissues of parasite location.

In conclusion, this study has demonstrated a pharmacokinetic and pharmacodynamic interaction between the synthetic anthelmintic ABM and R-CNE. Despite the limitations described, the integration of *in vitro* assays, *in silico* analysis, and *in vivo* pharmacoparasitological studies is relevant for the knowledge of the combinations of bioactive natural products and synthetic anthelmintics. Considering the increasing resistance of gastrointestinal nematodes to the different drug families, the development of new pharmacological tools is critical for the design of future successful strategies for parasite control.

## Data Availability Statement

The original contributions presented in the study are included in the article/Supplementary Material, further inquiries can be directed to the corresponding author.

## Ethics Statement

Animal procedures and management protocols were carried out according to internationally accepted animal welfare guidelines (AVMA, 2007) and approved by the Animal Welfare Committee of the Faculty of Veterinary Medicine, Universidad Nacional del Centro de la Provincia de Buenos Aires, Tandil, Argentina (Internal Protocol: FCV-UNCPBA 11/2018; approval date: August 27, 2018).

## Author Contributions

MM: Acquisition of data, analysis, and interpretation of data, drafting the manuscript. SL: Acquisition of data on the field. PC and ML: Acquisition of data on the field. DS and AS: *In silico* evaluation. LC-J: Contribution to conception and design, critically revising the manuscript. GV: Contribution to conception, design, and acquisition of data, critically revising the manuscript. AL: Contribution to conception, design and acquisition of data, analysis and interpretation of data, drafting and critically revising the manuscript. All authors have read and approved the final manuscript.

## Conflict of Interest

The authors declare that the research was conducted in the absence of any commercial or financial relationships that could be construed as a potential conflict of interest.

## Supplementary Material

Supplementary Figure S1 Docking analysis visualization of **(A)** Cel-Pgp-1 binding with ABA (yellow surface) and RCV (green surface). Binding site visualization of Cel-Pgp-1 in complex with **(B)** ABA (yellow sticks) and **(C)** R-CNE (red sticks). Cel-Pgp-1 is represented in blue ribbons. The key residues predicted for Cel-Pgp-1 interaction with ligands were highlighted in red. Images were generated using pymol.
