# Supplementary figures and images for: Plant-Derived Compounds as a Tool for the Control of Gastrointestinal Nematodes: Modulation of Abamectin Pharmacological Action by Carvone
Source: Front Vet Sci. 2020 Dec 16;7:601750. doi: 10.3389/fvets.2020.601750 (PMC7772935; doi:10.3389/fvets.2020.601750)

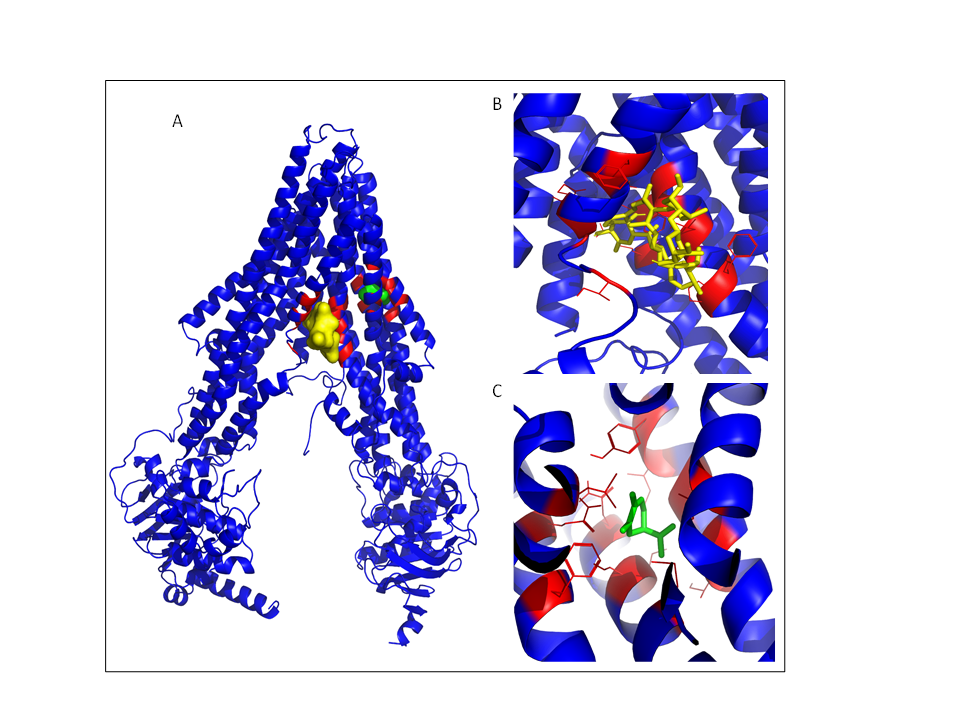

Supplement: Supplementary Figure S1 — Docking analysis visualization of (A) Cel-Pgp-1 binding with ABA (yellow surface) and RCV (green surface). Binding site visualization of Cel-Pgp-1 in complex with (B) ABA (yellow sticks) and (C) R-CNE (red sticks). Cel-Pgp-1 is represented in blue ribbons. The key residues predicted for Cel-Pgp-1 interaction with ligands were highlighted in red. Images were generated using pymol. [file Image_1.TIF]
